# DHHC3 interferes with antitumor immunity in melanoma cells

**DOI:** 10.18632/oncotarget.28880

**Published:** 2026-06-08

**Authors:** Chandan Sharma, Soonyean Hwang, Qingshi Liu, Martin E. Hemler

**Affiliations:** ^1^Department of Cancer Immunology and Virology, Dana-Farber Cancer Institute, Boston, MA 02215, USA; ^2^Current address: UCB, 87 Cambridge Park Dr, Cambridge, MA 02140, USA; ^3^Current address: Chinese Institutes for Medical Research, Beijing 100069, China

**Keywords:** oxidative stress, DHHC3, anti-cancer immunity, palmitoylation, melanoma

## Abstract

The protein-acyltransferase DHHC3 supports a few different tumor malignancies, but mechanisms have been unclear. Here we report that DHHC3-null B16F10 melanoma cells showed markedly elevated oxidative stress and senescence, accompanied by diminished tumor growth within immunocompetent C57/BL6 mice, but not in immunodeficient NOD-SCID mice. These results suggest that absence of DHHC3 enhances innate and/or adaptive anti-melanoma immunity. Consistent with this, DHHC3-null melanomas contained elevated numbers of anti-tumor cells (M1 macrophages, NK, CD4^+^T, CD8^+^T), whereas pro-tumor cells (M2 macrophages, MDSCs) were diminished. Unexpectedly, DHHC3 ablation minimally affected experimental metastasis of cells injected into either immunocompetent C57/BL6 or immunodeficient NOD-SCID mice. We conclude that DHHC3 ablation does not fundamentally alter melanoma cell growth and invasion/metastasis (independent of the immune system) despite its effects on oxidative stress and senescence. However, DHHC3 does control primary melanoma growth by supporting anti-melanoma immunity.

## INTRODUCTION

Protein acyl transferase 3 (DHHC3), one of 23 “DHHC” family members [1], palmitoylates several proteins, including 22–28 antioxidant/redox regulatory proteins, which explains how DHHC3 controls intracellular oxidative stress [2]. DHHC3 has emerged as an attractive therapeutic target in cancer [3, 4]. Elevated expression correlates with diminished patient survival in multiple cancer types [5], and it adversely affects efficacy of multiple anti-cancer chemotherapeutic drugs [2]. In breast and prostate cancer, DHHC3 ablation diminished primary tumor xenograft growth by mechanisms likely involving elevated oxidative stress and senescence, accompanied by possible contributions from innate immune cells [5]. However, the relative contributions of immune dependent and independent mechanisms were not established. In this regard, oxidative stress and senescence are known to affect tumor cell behavior by both immune dependent and independent mechanisms [6–8]. Here we used immunocompetent and immunodeficient mouse models to assess the relative immune-dependent and -independent contributions of DHHC3 to tumor growth and metastasis. A melanoma model was chosen because, despite DHHC3 gene expression correlation with diminished melanoma patient survival [5], a definitive DHHC3 role in melanoma had not been shown. Also, melanoma may be especially sensitive to effects of elevated oxidative stress [9].

DHHC3-ablated melanoma cells showed significantly diminished primary tumor growth in immunocompetent mice, but not in immunodeficient mice. Metastasis was not significantly diminished in either type of mouse model, and *in vitro* cell proliferation was likewise undiminished. A negative immunoregulatory role for DHHC3 was further evidenced by recruitment of both adaptive and innate immune cells into DHHC3-null tumors. In conclusion, DHHC3 appears to support melanoma growth primarily by restricting both innate and adaptive anti-cancer immunity.

## RESULTS

### DHHC3 effects on oxidative stress and senescence in B16F10 cells

**Figure 1:**
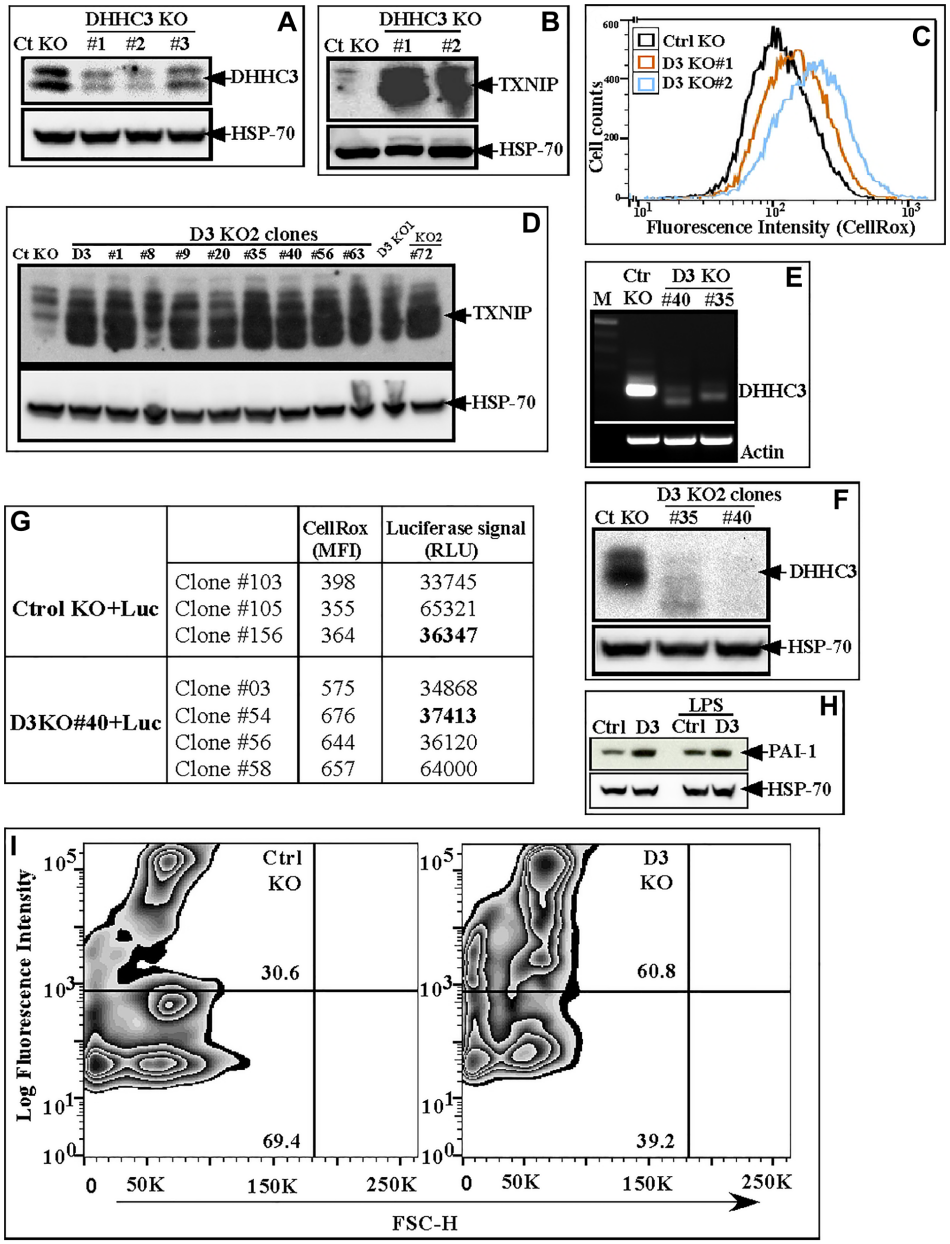
(**A**) DHHC3 protein in B16F10 cells (bulk population) with stably expressed CRISPR-Cas9 gRNAs (*#1, #2 and #3*) targeting DHHC3, and control gRNA (*Ctrl KO*); (**B**, **C**) TXNIP protein and CellRox signal (Oxidative stress) in Ct *KO* and D3 *KO#1* and *KO#2* B16F10 bulk populations; (**D**) TXNIP protein in the D3 KO1 bulk population plus 9 different single cell clones generated from the D3 *KO2* gRNA bulk population; (**E**, **F**) DHHC3 expression assessed by RT-PCR (E) and western blotting (F) in Ctrl KO and D3 KO clones *#35* and *#40*; (**G**) CellRox (MFI) and Luciferase signal (RLU) of Ctrl and D3 KO subclones selected after stable luciferase gene expression; (**H**, **I**) Expression of senescence markers, PAI-1 (LPS, treatment, lanes 3,4 serves as positive control for PAI-1 expression) (H) and SA-β-gal (I) in B16F10 Ctrl KO (#156) and D3 KO (#54) clones.

Three different gRNAs significantly reduced DHHC3 (D3) expression in B16F10 cells (Figure 1A). D3 knockout cells (obtained with gRNAs #1 and #2) displayed elevated oxidative stress (OS) as seen by elevated TXNIP (OS marker) expression and CellRox levels (Figure 1B, 1C). Results were confirmed using multiple single cell clones from D3 KO2 B16F10 population (Figure 1D, Supplementary Figure 1A). D3 KO clones #35 and #40 showed reduced gene (Figure 1E) and protein levels (Figure 1F) of DHHC3, due to deletions of 20 and 44 nucleotides respectively (Supplementary Figure 1B). PRED-TMR based protein topology analyses of D3 KO clone (#40) indicated deletions in transmembrane 2 (68-82 aa) of DHHC3. The ctrl KO and D3 KO (clone #40) B16F10 cells were further infected with lentiviral vector-expressing luciferase gene and then subcloned. D3 KO subclones (#03, #54, #56, #58) again displayed elevated oxidative stress/ROS (~1.5 to 1.9-fold) compared to Ctrl KO subclones (#103, #105 and #156) (Figure 1G, CellRox data). Based on similar luciferase expression (Figure 1G, RLU signal), ctrl KO clone #156 and D3 KO clone #54 were selected for subsequent experiments. Further assessment showed elevated expression (in D3 KO clone #54) of senescence markers, including plasminogen activator inhibitor 1 (Figure 1H), TNF-α (Supplementary Figure 1C), and SA-β-Gal (Figure 1I) in B16F10 cells.

### DHHC3 effects on B16F10 tumor growth

**Figure 2:**
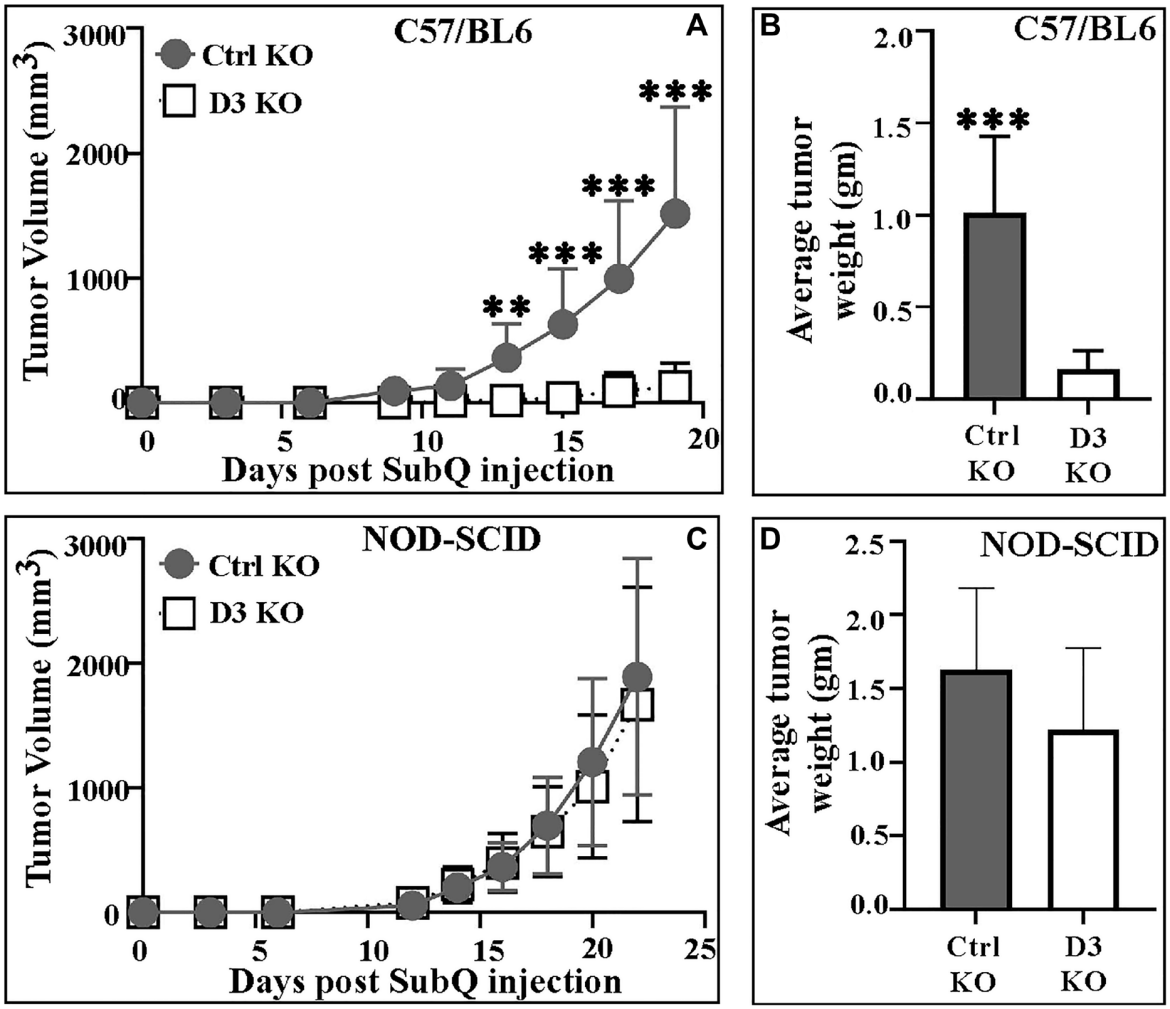
(**A**) Mean volumes for tumors derived from Ctrl KO (#156) and D3 KO (#54) B16F10 melanoma cells in C57/BL6 mice (*n* = 15 mice; ^***^P < 0.0001, ^**^P < 0.002); (**B**) Mean weights of Ctrl and D3 KO B16F10 tumors from C57/BL6 mice (*n* = 8 in ctrl; *n* = 6 in D3 KO group; ^***^P < 0.0004); (**C**) Mean volumes for tumors (Ctrl KO (#156); D3 KO (#54)) in NOD-SCID mice (*n* = 10 mice); (**D**) Mean weights of Ctrl and D3 KO tumors in NOD-SCID mice (*n* = 10, both groups; *P* = 0.11). All *P*-values were determined using the unpaired 2-tailed *T*-test. Tumor volume data for Ctrl KO and D3 KO tumors in each individual C57/BL6 and NOD-SCID mouse are shown in Supplementary Figure 2, and representative photo images are in Supplementary Figure 3A.

B16F10 melanoma cell clones, +/− DHHC3 ablation, showed essentially identical growth rates over 1–2 day intervals *in vitro* (data not shown). However, following subcutaneous (sub Q) injection into syngeneic immunocompetent C57/BL6 mice, D3 KO (clone #54), compared to ctrl KO cells, yielded tumors with significantly reduced volumes (Figure 2A; Supplementary Figure 2A, 2B, 3A), and weights (Figure 2B). A repeat experiment (each group *N* = 5) again showed significantly reduced growth for tumors derived from D3 KO cells (data not shown). By contrast, subcutaneous injection of D3 KO B16F10 cells into immunodeficient NOD-SCID mice yielded tumors not significantly reduced in either volume (Figure 2C; Supplementary Figure 2C, 2D, 3A) or weight (Figure 2D).

### DHHC3 ablation effects on B16F10 lung metastasis

To assess experimental lung metastasis, ctrl (#156) and D3 (#54) KO B16F10 cells were injected into the tail veins of both immunocompetent (C57/BL6) and immunodeficient (NOD-SCID) mice. Within C57/BL6 mice, D3 KO cells did not show reduced metastasis as assessed by HE staining (Supplementary Figure 3B, 3C). In fact, 3/10 mice showed a larger number of tumor colonies from D3 KO cells, compared to Ctrl KO cells. Within immunodeficient NOD-SCID mice, there was an insignificant reduction in lung metastasis due to DHHC3 ablation, as seen by luciferase signal (BLI) quantitation (Supplementary Figure 3D, 3E) and HE staining (Supplementary Figure 3F), in separate experiments.

### Elevated anti-tumor immune cell recruitment into DHHC3 KO primary tumors

**Figure 3:**
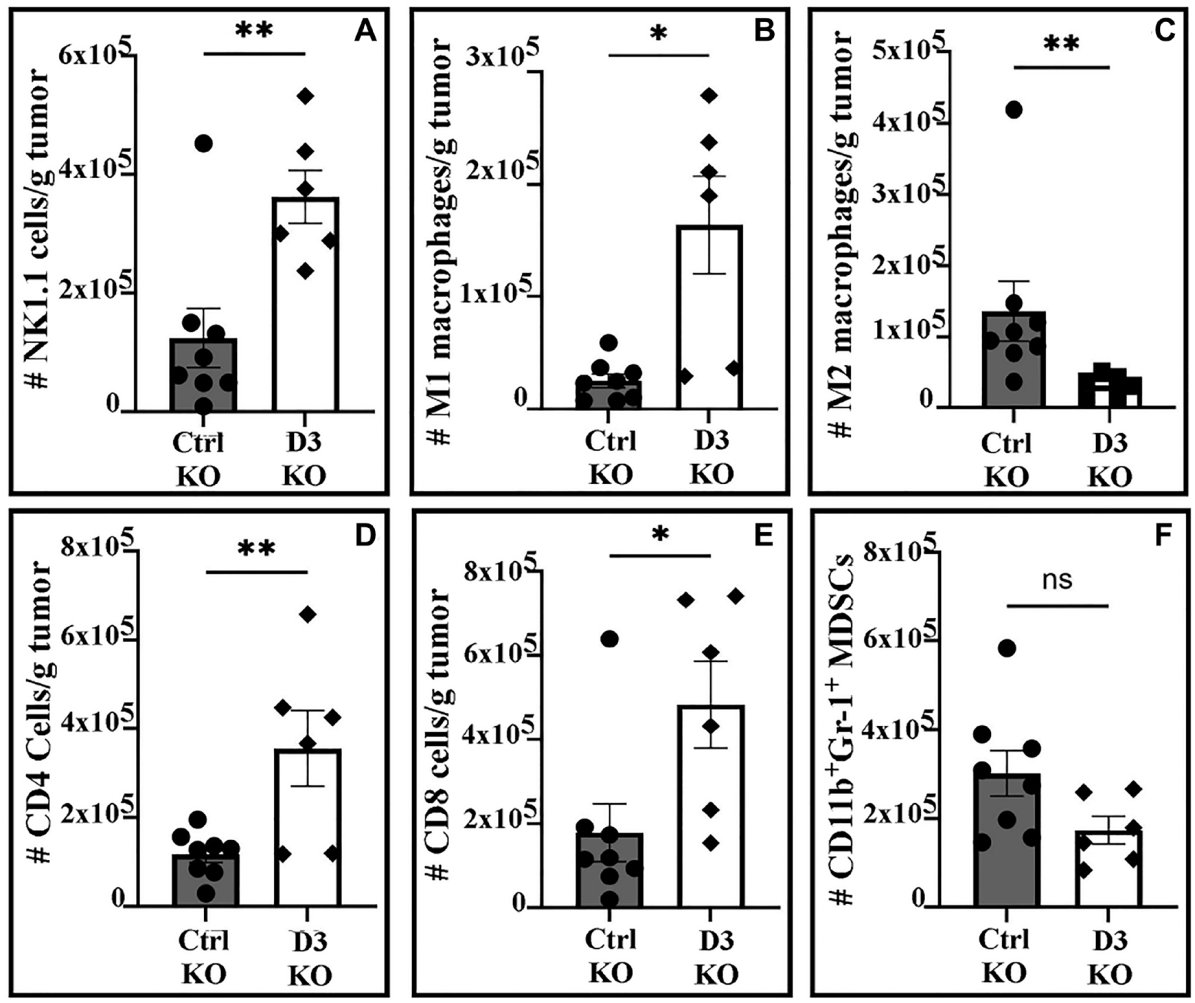
Immune cell numbers/gram of tumor from C57/BL6 mice. (**A**) NK cells; (**B**) M1 macrophages; (**C**) M2 macrophages; (**D**) CD4^+^ T cells; (**E**) CD8^+^ T cells, (**F**) MDSCs. Statistical comparisons were made using either the unpaired 2-sided *T*-test (A, D, F) or the Mann-Whitney test (B, C, E). ^*^P < 0.02, ^**^P < 0.008.

Ctrl KO and D3 KO primary tumors from C57/BL6 mice were analyzed (using flow cytometry) for immune cell recruitment. Significantly higher numbers of NK cells (*P* < 0.005), M1 macrophages (*P* < 0.01), CD4^+^ and CD8^+^ T cells (*P* < 0.008 and 0.02 respectively) were observed in D3 KO xenograft tumors as compared to ctrl KO tumors (Figure 3A, 3B, 3D, 3E). Conversely, numbers of pro-tumor M2 macrophages and MDSCs were significantly (*P* < 0.004, Figure 3C) or marginally (*P* = 0.07, Figure 3F) reduced in D3 KO tumors. There were also slight differences in numbers of CD19 B cells (*P* = 0.16), neutrophils (*P* = 0.14) and CD4^+^ Tregs (*P* = 0.15), consistent with increased anti-cancer immunity, but these were not significant. Dendritic cell populations (cDC1, *P* = 0.9; cDC2, *P* = 0.46) showed minimal differences (Supplementary Figure 4A–4E).

## DISCUSSION

### Immune-dependent effects

Although DHHC3 has been suggested to support tumor growth, and metastasis [2, 5], it was not clear whether effects were dependent or independent of anti-cancer immunity. Using a melanoma model, we establish that DHHC3 ablation significantly diminishes primary tumor growth selectively, in immunocompetent, but not immunodeficient mice. Consistent with this, we observed selective recruitment of anti-cancer immune cells (NK, M1 macrophages, CD4T, CD8T) to DHHC3-null tumors, coupled with selective reduction in pro-tumor cells (M2 macrophages, MDSCs). Although we did not analyze specific effector molecules in this study, we showed previously that elevated innate anti-cancer immunity, upon DHHC3 ablation, was accompanied by an upregulated chemokine pattern consistent with a senescence-associated secretory phenotype (SASP) response, which is known to trigger innate immune cell recruitment [5].

In B16F10 melanoma cells, DHHC3 ablation led to elevated oxidative stress (increased TXNIP expression, higher CellROX signal) and senescence (elevated senescence markers SA-β-gal, PAI-1, TNF-α). In a prior study (involving breast cancer cells), we showed that TXNIP ablation significantly reversed effects of ZDHHC3 ablation on oxidative stress and subsequent oxidative-stress dependent events, including senescence associated chemokine secretion [5]. Also, we showed that anti-oxidants can reverse oxidative stress-dependent events arising subsequent to DHHC3 ablation [5]. Furthermore, elevated innate anti-cancer immunity upon DHHC3 ablation was accompanied by an upregulated chemokine pattern consistent with a senescence-associated secretory phenotype (SASP) response [5]. Elevated oxidative stress and senescence are well established to lead to a SASP response, which typically results in selective chemokine secretion and facilitates recruitment of both innate and adaptive anti-tumor immune cells, leading to clearance of senescent cells and tumor growth inhibition [8, 10].

It was suggested elsewhere that DHHC3 might inhibit adaptive immunity by directly palmitoylating PD-L1 [11], a negative regulator of adaptive anti-cancer immunity [12]. However, we did not observe PD-L1 to be palmitoylated in tumor cells, regardless of DHHC3 presence (unpublished results) and PD-L1 is listed as not palmitoylated in the Swiss-PALM database [13]. An intriguing alternative possibility is that DHHC3 palmitoylation of CMTM6 [2], which is needed to support PD-L1 expression [14, 15], could contribute to DHHC3 effects on adaptive immunity.

### Immune-independent effects

Multiple studies show that elevated oxidative stress and senescence can result in reduced tumor growth by immune-independent mechanisms [6, 16–17], but our data is not consistent with those observations. B16F10 melanoma cell clones, +/− DHHC3 ablation, showed essentially identical *in vitro* growth rates over 1–2 day intervals. Perhaps more surprisingly, DHHC3 ablation did not cause significant alteration in B16F10 primary tumor growth in immunodeficient NOD-SCID mice. These results, coupled with absence of changes in tumor cell proliferation, provide assurance that DHHC3 ablation has not fundamentally altered intrinsic cell growth-related physiology. In breast cancer cells, DHHC3 ablation did not affect cell proliferation *in vitro* (up to 72 hr) or 3D soft agar growth (through 14 days, despite compelling genetic changes associated with oxidative stress and senescence [5]. We suspect that elevated oxidative stress and senescence in melanoma cells may not reach the threshold needed to directly affect primary tumor growth, unless a fully competent immune system is appropriately triggered (i.e., through a SASP-type response).

### Effects on metastasis

Metastasizing melanoma cells appear to be particularly sensitive to elevated oxidative stress [9]. DHHC3 levels are elevated in human metastatic breast cancer samples and DHHC3 ablation markedly diminished breast cancer metastasis [5]. Hence, it was unexpected that DHHC3-ablated B16F10 melanoma cells did not show significantly decreased metastasis in either immune-deficient or immunocompetent mice. Again, oxidative stress may not have reached the necessary threshold. Additionally, enhanced ROS and senescence are known to impact cell behavior differently in different microenvironments [18, 19]. Our findings that DHHC3 ablation did not significantly affect metastatic growth, in terms of either colony number or size, after tail vein injection into either immunocompetent or immunocompromised mice (Supplementary Figure 3), further emphasize that fundamental growth-related physiology was unchanged in the melanoma cells.

### Role of DHHC3 in melanoma

We show here that tumor growth in a B16F10 tumor model is significantly diminished, within an immunocompetent microenvironment, due to DHHC3 ablation. These results are likely relevant to human melanoma because human melanoma is particularly sensitive to augmented anti-cancer immunity [20] and elevated DHHC3 gene expression correlates with diminished survival in human melanoma [5]. Furthermore, it was shown elsewhere that DHHC3 palmitoylates tyrosinase [21], which plays a key role in melanoma progression [22].

## MATERIALS AND METHODS

### Antibodies and reagents

DHHC3 (ab31837) pAb and TXNIP (K0205-3) mAb were from Abcam and MBL international, respectively. Anti-mouse PAI-1 and TNF-α antibodies were from senescence associated secretory phenotype (SASP) antibody sampler kit (Cell Signaling; 85741). CD45 (103149), F4/80 (123108), CD11b (108706), CD11c (117348), CD25 (101918), CD64 (139309), CD8α (100742), NK1.1 (108710), TCRγδ (118124), TCRβ (109251) and FoxP3 (126419) antibodies were from Biolegend; CD3 (BD741788), CD4 (BD563790), CD19 (BD612781), CD24 (BD562477) and CD69 (BD553237) antibodies were from BD Biosciences. CellRox and C12FDG kits were from Invitrogen. B16F10 cells were from ATCC and cultured in DMEM media (Invitrogen) containing 10% FBS (Sigma), HEPES and 1% penicillin-streptomycin (Invitrogen) at 37^o^C in humidified 5% CO_2_ incubator. Mycoplasma was tested using MycoAlert kit (Lonza Biologics).

### Generation of knockout cells

DHHC3-null B16F10 cells were generated by infection with lentiviral particles containing DHHC3 gRNA and Cas9, as described [23]. DHHC3 gRNA#1 (5′-CACCGTAAGCGGTGCATTCGCAAGA-3′), #2 (5′-CACCGCTGTACGCGTAGTCTCGGGA-3′) and #3 (5′-CACCGTGATGCTGTACGCGTAGTCT-3′) were custom synthesized by IDT, Coralville, USA. Nonspecific control gRNA was from TC Cheong [24]. Control and DHHC3 KO cells were subcloned by serial dilution using 96 well plates.

### Protein and cellular assays

Assays for oxidative stress (CellRox), senescence (C12FDG dye), mRNA (Qiagen one-step RT-PCR kit 210212), and protein (western blotting) were as described [5]. Regarding western blotting, protein Kaleidoscope MW markers (Cat #1610375) do not emit a chemiluminescent signal and thus are not detected by X-ray film. Instead, the nylon or nitrocellulose membranes are precisely aligned with the X-ray film immediately following development, such that the positions of the MW markers can be clearly marked on the film. *In vitro* cell proliferation was assessed by monitoring cell numbers over a 24–48 hr time period.

### *In vivo* tumor experiments

Tumor growth was assessed as described [5]. Briefly, 1 × 10^6^ B16/F10 cells (+D3) were injected subcutaneously into 4–6 weeks old female C57BL/6 (Strain 000664, Jackson Labs) and NOD-SCID (Strain 394, Charles River) mice. Tumors were measured every other day and volume was calculated by “length × width^2^ × 0.52”.

### Tail vein lung metastasis

B16F10 cells (+/−D3) were injected into lateral tail veins of C57BL6 and NOD-SCID mice using a 27-gauge needle. After two weeks, lungs were isolated, fixed in 10% (v/v) neutral buffered Formalin solution (36 hrs), were washed and dehydrated in 70% ethanol solution, processed into paraffin sections, then hematoxylin and eosin (HE) stained. In a separate experiment with NOD-SCID mice, D-luciferin (Caliper Life Sciences, 150 mg/kg) was injected 10 minutes prior to capturing images (IVIS Lumina-II system, PerkinElmer, Waltham, MA; analyzed using Living Image 4.2 software). Signal intensity was calculated as sum of photon counts within the region of interest minus background luminescence. Lungs from these mice were HE stained to quantify tumor colonies. Luciferase expression in KO B16F10 subclones (+D3) was measured *in vitro* using Bio-GLO Luciferase Assay reagent and POLARstar Omega instrument from BMG Labtech.

### Immune cell subset flow analyses

Single cell suspensions from Ctrl and D3 KO tumors were prepared using Mouse Tumor Dissociation kit (Cat #130-096-730; Miltenyi Biotec Inc; Auburn, CA, USA), as described [25]. Isolated cells were stained with Zombie fixable viability dye (Biolegend), then with antibodies to specific immune cell subsets (in 1X PBS buffer, 1% FBS, 1 mM EDTA). Cells were analyzed using Fortessa X20 (BD) cytometer and R statistical programming language.

### Statistical analyses

Statistical analyses were performed using either the unpaired 2-tailed *T*-test or Mann-Whitney test as indicated in Figure Legends.

## CONCLUSIONS

We show that DHHC3 ablation leads to diminished melanoma primary growth, by a mechanism involving elevated innate and/or adaptive anti-cancer immunity, likely triggered by increased oxidative stress and senescence. However, elevated oxidative stress and senescence alone did not impair either primary tumor growth in immunodeficient NOD-SCID mice, or metastasis (in either immunocompetent C57BL6 mice or NOD-SCID mice). Together these results provide firm evidence for DHHC3 having an immunoregulatory role and further support the potential of DHHC3 as a target for anti-cancer therapy in melanoma. Furthermore, elevated anti-cancer immunity may likely explain diminished tumor growth not only for DHHC3-ablated melanoma (this study) and breast cancer tumors [5], but also prostate cancer cells [2]. Also, it is feasible to assume that diminished anti-cancer immunity could explain poor outcomes for human breast, melanoma and prostate patients with elevated DHHC3 gene expression [2, 5].

## SUPPLEMENTARY MATERIALS


