# Supplementary material for: DHHC3 interferes with antitumor immunity in melanoma cells
Source: Oncotarget. 2026 Jun 8;17:306–13. doi: 10.18632/oncotarget.28880 (PMC13249276; doi:10.18632/oncotarget.28880)
Supplement: Supplementary file 1 [file oncotarget-26-049662-s001.pdf]

DHHC3 interferes with antitumor immunity in melanoma cells

SUPPLEMENTARY MATERIALS

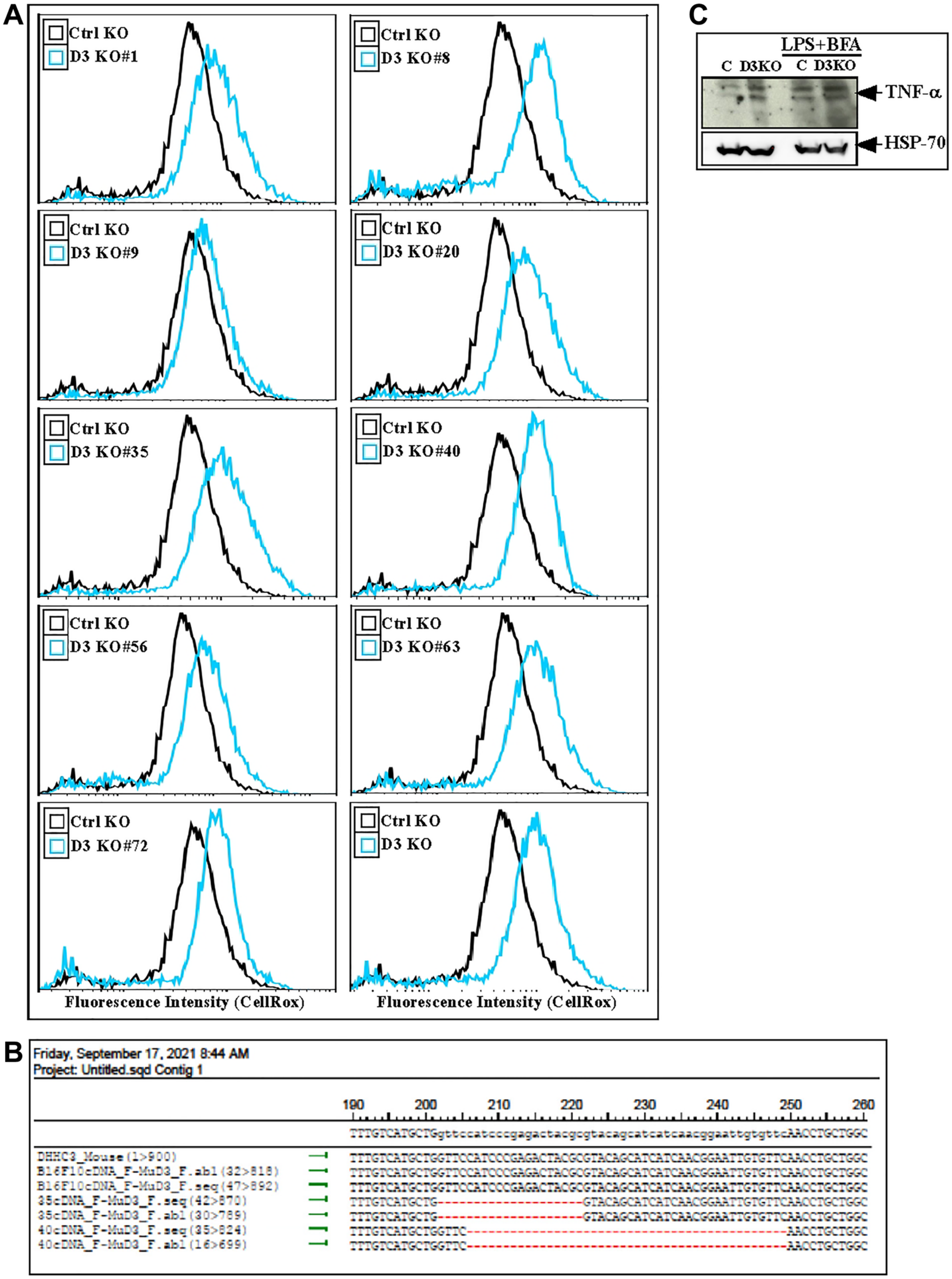

Supplementary Figure 1: (**A**) CellRox signal (MFI) of cells cloned from D3 KO2 gRNA population; (**B**) Alignment of Ctrl and D3 KO DHHC3 DNA sequences (clones #35, #40). (**C**) mouse TNF-α (senescence marker) protein in Ctrl and D3 KO clones (LPS+BFA treatment, lanes 3,4 serves as positive control for TNF-α expression) LPS, Lipopolysaccharide and BFA, Brefeldin A.

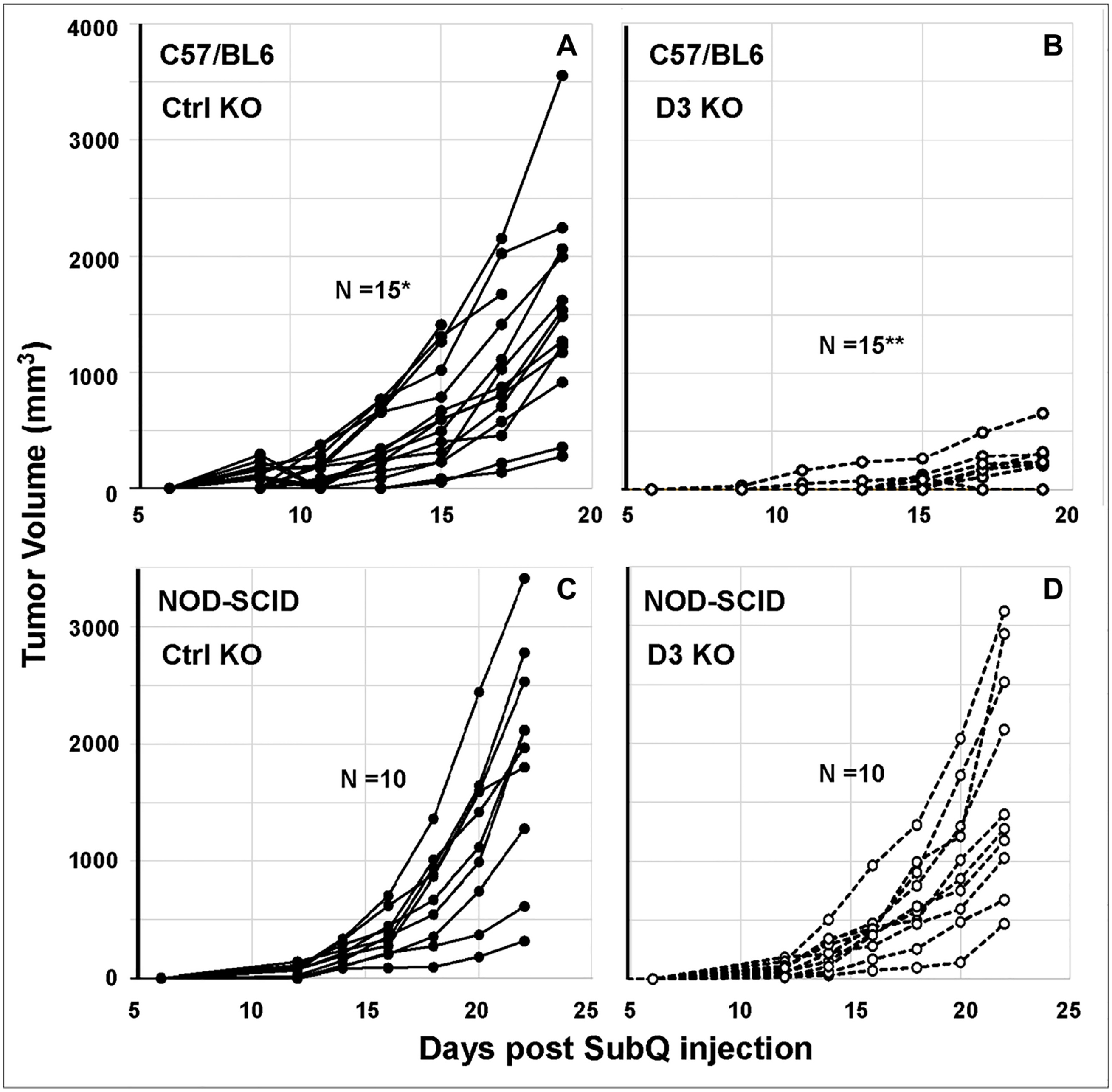

Supplementary Figure 2: (**A**) Volumes for Ctrl KO tumors in individual C57/BL6 mice. $N = 15^{*}$, note that data points are missing at 19 days (for 2 mice) and 17 days (for one mouse); (**B**) Volumes for D3 KO tumors in individual C57/BL6 mice. $N = 15^{**}$, note that 8 of the 15 mice showed no detectable tumor growth. (**C**) Volumes for Ctrl KO tumors in individual NOD-SCID mice, *N* = 10; (**D**) Volumes for D3 KO tumors in individual NOD-SCID mice, *N* = 10.

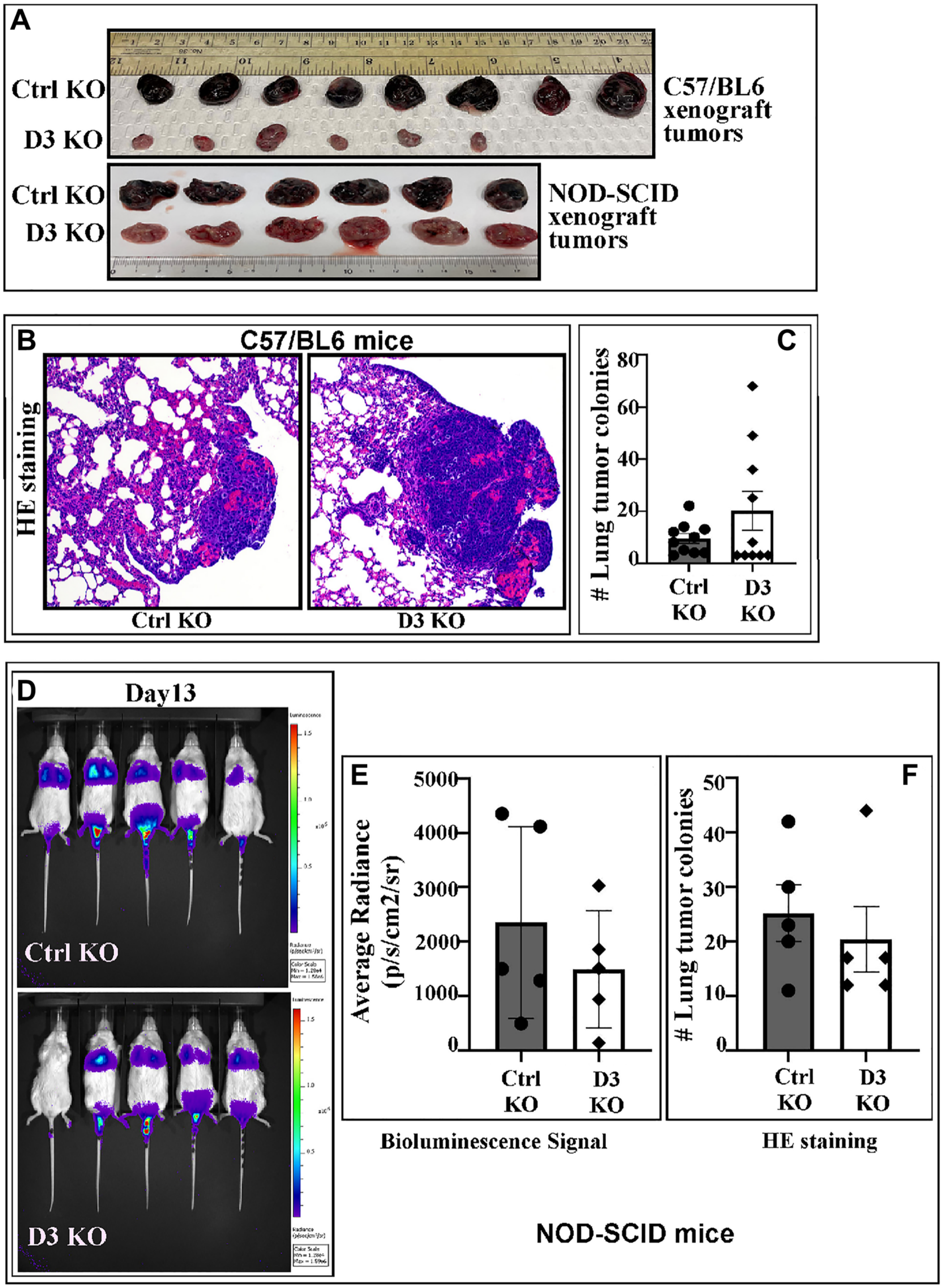

Supplementary Figure 3: (**A**) Tumors from Ctrl and D3 B16F10 cells in C57BL6 mice (upper panel), and NOD-SCID mice (bottom panel); (**B**) HE staining of lung sections showing metastatic colonies from Ctrl and D3 KO B16F10 cells injected into C57/BL6 mice; (**C**) Mean tumor colony numbers (HE staining) from both groups ($N = 10$) in C57/BL6 mice ($P = 0.18$); (**D**) Bioluminescence (BLI) images of Ctrl (upper) and D3 KO (lower) NOD SCID mice, Day 13 post tail vein injection; (**E**) Mean radiance (BLI signal, photons/second/cm^2/steradian) of lungs from Ctrl and D3 KO NOD SCID mice ($n = 5$; $P = 0.38$); (**F**) Mean tumor colony numbers (from HE staining) in NOD-SCID mice ($n = 5$; $P = 0.56$). Statistical analysis (in C and D) was performed using the unpaired 2-tailed *T*-test.

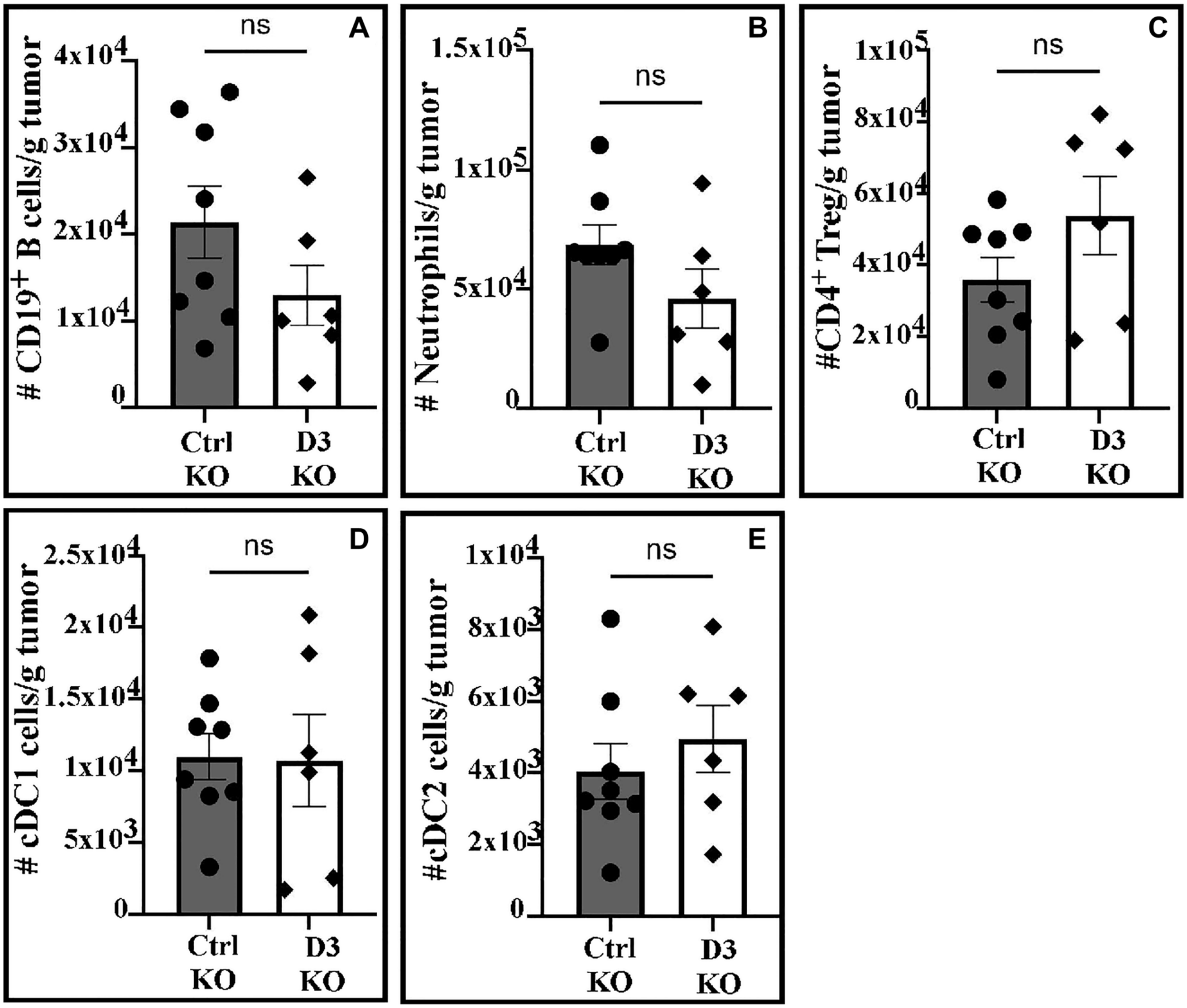

Supplementary Figure 4: Immune cell numbers/gram of tumor from C57/BL6 mice. (**A**) CD19+ B cells; (**B**) neutrophils; (**C**) CD4+ Treg; (**D**) cDC1; (**E**) cDC2. Statistical analysis was performed using the unpaired 2-tailed *T*-test (A–E).
